# Open Development and Clinical Validation of Multiple 3D-Printed Nasopharyngeal Collection Swabs: Rapid Resolution of a Critical COVID-19 Testing Bottleneck

**DOI:** 10.1128/JCM.00876-20

**Published:** 2020-07-23

**Authors:** Cody J. Callahan, Rose Lee, Katelyn E. Zulauf, Lauren Tamburello, Kenneth P. Smith, Joe Previtera, Annie Cheng, Alex Green, Ahmed Abdul Azim, Amanda Yano, Nancy Doraiswami, James E. Kirby, Ramy A. Arnaout

**Affiliations:** a Department of Radiology, Beth Israel Deaconess Medical Center, Boston, Massachusetts, USA; b Clinical Microbiology Laboratories, Division of Clinical Pathology, Department of Pathology, Beth Israel Deaconess Medical Center, Boston, Massachusetts, USA; c Division of Infectious Disease, Department of Medicine, Beth Israel Deaconess Medical Center, Boston, Massachusetts, USA; d Harvard Medical School, Boston, Massachusetts, USA; e Division of Urologic Surgery, Department of Surgery, Beth Israel Deaconess Medical Center, Boston, Massachusetts, USA; f Division of Respiratory Therapy, Beth Israel Deaconess Medical Center, Boston, Massachusetts, USA; g Department of Medicine, Beth Israel Deaconess Medical Center, Boston, Massachusetts, USA; h Division of Perioperative Services, Department of Central Processing, Beth Israel Deaconess Medical Center, Boston, Massachusetts, USA; i Division of Infection Control/Hospital Epidemiology, Silverman Institute for Healthcare Quality and Safety, Beth Israel Deaconess Medical Center, Boston, Massachusetts, USA; j Division of Clinical Informatics, Department of Medicine, Beth Israel Deaconess Medical Center, Boston, Massachusetts, USA; Rhode Island Hospital

**Keywords:** COVID-19, SARS-CoV-2, virological testing, diagnostic testing, epidemiology

## Abstract

The pandemic caused by severe acute respiratory syndrome coronavirus 2 (SARS-CoV-2) has caused a severe international shortage of the nasopharyngeal swabs that are required for collection of optimal specimens, creating a critical bottleneck blocking clinical laboratories’ ability to perform high-sensitivity virological testing for SARS-CoV-2. To address this crisis, we designed and executed an innovative, cooperative, rapid-response translational-research program that brought together health care workers, manufacturers, and scientists to emergently develop and clinically validate new swabs for immediate mass production by 3D printing. We performed a multistep preclinical evaluation of 160 swab designs and 48 materials from 24 companies, laboratories, and individuals, and we shared results and other feedback via a public data repository (http://github.com/rarnaout/Covidswab/). We validated four prototypes through an institutional review board (IRB)-approved clinical trial that involved 276 outpatient volunteers who presented to our hospital’s drive-through testing center with symptoms suspicious for COVID-19. Each participant was swabbed with a reference swab (the control) and a prototype, and SARS-CoV-2 reverse transcriptase PCR (RT-PCR) results were compared. All prototypes displayed excellent concordance with the control (κ = 0.85 to 0.89). Cycle threshold (*C_T_*) values were not significantly different between each prototype and the control, supporting the new swabs’ noninferiority (Mann-Whitney U [MWU] test, *P* > 0.05). Study staff preferred one of the prototypes over the others and preferred the control swab overall. The total time elapsed between identification of the problem and validation of the first prototype was 22 days. Contact information for ordering can be found at http://printedswabs.org. Our experience holds lessons for the rapid development, validation, and deployment of new technology for this pandemic and beyond.

## Abstract (precis)

The pandemic caused by severe acute respiratory syndrome coronavirus 2 (SARS-CoV-2) has caused a severe international shortage of the nasopharyngeal swabs that are required for collection of optimal specimens, creating a critical bottleneck blocking clinical laboratories’ ability to perform high-sensitivity virological testing for SARS-CoV-2. To address this crisis, we designed and executed an innovative, cooperative, rapid-response translational-research program that brought together health care workers, manufacturers, and scientists to emergently develop and clinically validate new swabs for immediate mass production by 3D printing.

## INTRODUCTION

**FIG 1:**
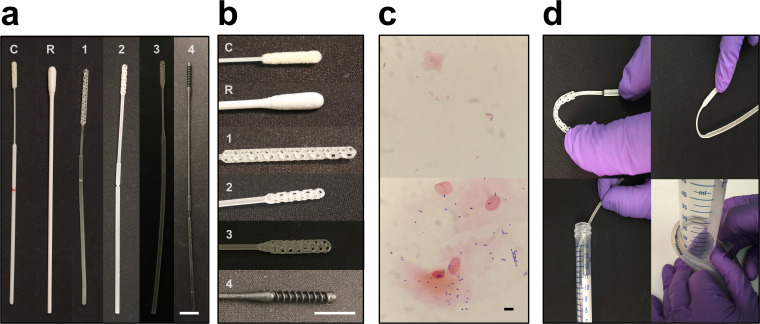
Control and prototype swabs. (a) From left to right, the control swab (C; Copan 501CS01), a repurposed urogenital cleaning swab approved for NP testing through our process (R), prototype 1 (Resolution Medical), prototype 2 (EnvisionTec), prototype 3 (Origin.io), and prototype 4 (HP, Inc.). (b) From top to bottom, close-ups of the heads of the swabs in panel a. Bars, 1 cm. (c) Examples of Gram stains of cheek swabs using control (top) and prototype (bottom) swabs. Bar, 10 μm. (d) Examples of materials testing. Clockwise from top left, head flexibility and robustness to fracture, neck flexibility and robustness to fracture, robustness to repeat insertion into and removal from a tortuous canal (diameter, 3 cm), and break point evaluation.

Since the emergence of the coronavirus disease 2019 (COVID-19) pandemic, more than 2.5 million cases have been diagnosed worldwide (1). These diagnoses were made using material collected with nasopharyngeal swabs, which provide the highest sensitivity for detecting severe acute respiratory syndrome coronavirus 2 (SARS-CoV-2) infection during early infection using commercial reverse transcriptase PCR (RT-PCR)-based assays (2). A nasopharyngeal (NP) swab is an FDA class I exempt medical device roughly 15 cm in length and 2 to 3 mm in diameter designed to collect secretions from the posterior nasopharynx (Fig. 1a, left, and Fig. 1b, top). The head of the swab is generally coated with short synthetic filaments called flock or with spun fibers. The swab is inserted into the nasopharynx, rotated several times to collect material, and then placed in a vial containing a few milliliters of transport medium. A break point on the shaft enables detachment and release of the head into the vial, which is then sealed and sent for testing.

The rapid spread of SARS-CoV-2 has resulted in severe shortages of NP swabs, due to both manufacturing stoppages, resulting in decreased supply, and the spread of the pandemic, resulting in unprecedented demand (3). To address the swab shortage, hospitals and other testing centers have repurposed other commercially available swabs (e.g., throat and urogenital) to collect nasal epithelial cells for testing (Fig. 1a, second from the left, and Fig. 1b, second from the top). However, such swabs are suboptimal for swabbing the nasopharynx due to differences in size and flexibility and the possibility that the materials of which they are made may inhibit PCR (4, 5). Material from other anatomical sites is being investigated for its ability to be substitutes for nasopharyngeal samples, but reports are still preliminary (6–8).

One solution to the swab crisis is to design and 3D print swabs. Advantages of 3D printing include simplicity (avoiding the multistep process of applying flock), the widespread availability of 3D printing capacity, and the ability to iterate prototypes rapidly (9). To resolve the swab shortage crisis, we have been coordinating an open collaborative process that has brought together many medical centers, individuals, academic laboratories, and both new and well-established manufacturers (10). As part of this process, we have been testing and continuously providing feedback on prototype swabs in order to proceed rapidly but safely toward the development of swabs that can be used clinically, at volumes equal to the need. The openness of the process was a conscious choice supported by a substantial body of scientific literature, including our experience, that demonstrates the advantages of openness over closed or hybrid approaches (11–13). At our institution, this process has led to an ongoing clinical trial of several prototype swabs, the first results of which we report here.

## MATERIALS AND METHODS

### Process.

Our goal was to quickly develop and clinically validate NP swabs that could be mass produced and made available for testing as soon as possible. We created a public repository using GitHub, a company that provides free hosting for collaborative projects on the Web, most often used by programmers to share, comment on, and codevelop computer code (http://www.github.com/rarnaout/Covidswab) (10). We provided a clear description of the problem and updated the repository with what we learned, and we encouraged others to do the same. By tapping our personal and professional networks, we assembled an *ad hoc* network of manufacturers that included companies, academic groups, and individuals. This grew to include other medical centers interested in helping develop and test new swabs. These other groups were given the ability to add to the repository as desired; to date, the greatest number of contributions has been made by us.

We devised a three-phase process consisting of preclinical evaluation (phase I), production considerations (phase II), and field testing (phase III). We described these processes on the repository for all to see. We took high-resolution photographs of all swab prototypes and stored phase I results in a Microsoft Excel (Microsoft Corporation, Redmond, WA, USA) spreadsheet that remains publicly available in the repository. All contributors could see each other’s designs and our feedback and generated new designs accordingly.

We made our personal contact information freely available to facilitate communication and speed the delivery of prototypes. We involved representatives of our institution’s nursing, legal, intellectual property, leadership, purchasing, human resources, communications, and contracting teams and the institutional review board (IRB) early and often in order to facilitate open development, reassign idled staff to our process, and minimize lead times during the rapidly changing situation.

### Phase I: preclinical evaluation.

**(i) Design.** An infectious disease physician, a clinical pathologist (clinical microbiologist), and a respiratory therapist tested each prototype swab for design and mechanical properties (Fig. 1c and d). These included size measurements of the head, neck, shaft, and break point (requirement of ∼15 cm to reach the posterior nasopharynx; head diameter of 1 to 3.2 mm to pass into the midinferior portion of the inferior turbinate and be able to maneuver appropriately without catching on anatomical variants such as septal spurs or a deviated nasal septum); surface properties, such as smoothness (with roughness leading to an unpleasant feel and risk of bleeding); flexibility versus brittleness of the head, neck, shaft, and break point (to avoid fracture during use); durability (e.g., ability to tolerate 20 rough repeated insertions into a 4-mm-inner-diameter clear plastic tube curved back on itself with a curve radius of ∼3 cm; ability of tip and neck to be bent 90° without breaking; ability to revert to initial form following bend of 45°) (Fig. 1d); strength (to resist breakage under rough but reasonable manipulation); and other factors as applicable (e.g., stickiness and smell) (Table 1).

**TABLE 1:** Preclinical (phase I) evaluation testing parameters and acceptance criteria

Parameter	Description or comments	Acceptance criteria
Measurements		
Total length	Length of NP swab from end to end	15–16 cm
Head length	Length of NP swab head used for collection of secretions and cellular material from posterior nasopharynx	1.5–3.5 cm
Head diam	Diam of NP head allowing passage into posterior nasopharynx; must be sufficiently small for passage beyond inferior turbinate without catching on abnormal anatomy, such as septal spurs or a deviated nasal septum, but must otherwise maximize surface area for specimen collection	1–4 mm
Neck diam	A neck thinner than the head and shaft allows flexibility, easing manipulation of the swab in the posterior nasopharynx	1–2 mm
Neck length	Length of neck following the head tip prior to the shaft	3–3.5 cm
Break point location	A break point is a scoring or narrowing that allows the user to break the head off into the viral transport tube. This must be sufficiently easy that breaking can occur without need of, e.g., scissors and without excessive infection risk but not so easy as to risk breaking during insertion into the patient. Distance from head tip to break point must be less than the length of the tube (Fig. 1d, bottom left).	7–10 cm
Surface properties		
Smoothness	Swabs should be sufficiently smooth to touch and minimally abrasive for patient comfort and safety. In particular, the tip should not be sharp, so as to prevent puncture injuries and minimize epistaxis risk.	Sufficient smoothness
Adhesiveness or residue	Swabs should not feel sticky or tacky or leave a residue behind with handling, as such residue could in principle have unwanted effects.	Not sticky
Odor	Swabs should not have an unusual chemical or metallic odor that could be an allergen or safety hazard to patients.	Must have a tolerable odor (e.g., no odor or very faint “plastic smell” is acceptable; strong, acrid, or glue smell is unacceptable)
Mechanical properties		
Head and neck flexibility	Swabs must be flexible enough to be maneuvered into the posterior nasopharynx	Ability to bend head and, separately, neck at least 90 degrees without detachment. Ability of swab neck to revert to initial form following repetitive bending to 45° in both directions 45 times (Fig. 1d, top)
Durability/strength	Swabs must be durable enough to not break with reasonable manipulation	Ability to tolerate 20 rough repeated insertions into a 4 mm-inner-diam clear plastic tube curved with a radius of 3 cm (Fig. 1d, lower left)
Additional factors		
Collection sufficiency	Swabs must be able to collect sufficient material for detection of viral nucleic acid. Collection sufficiency was approximated by Gram staining of an interior cheek swab compared to standard Copan swab (model 501CS01) as a control.	At least 10 clusters of bacteria/cells at ×40 magnification (Fig. 1c)
PCR compatibility	Swabs must not inhibit PCR	Swab material was incubated in standard viral transport medium overnight, spiked with 2× the limit of detection (200 copies/ml) of the SARS-CoV-2 amplicon target, and tested using the Abbott RealTime SARS-CoV-2 assay on the Abbott m2000 platform.

**(ii) Collection sufficiency.** We assessed the ability to collect sufficient material for testing using Gram staining of a swab of the interior cheek smeared onto a standard microscopy slide as a surrogate for NP swabbing and comparison to Gram stain of a swab of the interior cheek using Copan Diagnostics, Inc. (Mantua, Italy), model 501CS01 (FLOQSwab) as the control (Fig. 1c). Cheek swabbing was performed instead of NP swabbing as the least invasive and most readily available source of secretions, making it possible to test head designs even for prototypes that were deemed inappropriate as NP swabs. Slides were heat fixed and Gram stained according to the BD BBL Gram stain test kit protocol (14). Slides were examined at ×40 magnification for the presence of both epithelial cells and bacteria. Prototypes were passed if the amounts of bacteria and epithelial cells were qualitatively similar to those of the control (which contained multiple bacteria and epithelial cells per high-power field).

**(iii) PCR compatibility.** We tested PCR compatibility by placing the swab head-downward after breaking it off at the break point, when present (as in a typical NP swab collection), in 3 ml of modified CDC VTM (Hanks’ balanced salt solution containing 2% heat-inactivated fetal bovine serum [FBS], 100 μg/ml gentamicin, 0.5 μg/ml amphotericin B [Fungizone], and 10 mg/liter phenol red [15]) overnight to allow any PCR-inhibitory material to leach into the medium, spiking 1.5 ml with 200 copies/ml of control SARS-CoV-2 amplicon target (representing 2 times the limit of detection on our system), vortexing, and testing using the Abbott RealTime SARS-CoV-2 assay on an Abbott m2000 RealTime system platform (16), following the same protocol as for clinical testing (37 cycles, with a cycle threshold [*C_T_*] of ≤31.50 being reported as positive). PCR-positive prototypes passed.

### Phase II: production considerations.

We considered stability to autoclaving by repeating phase I testing on postautoclaved materials, manufacturers’ short-term strategies for individual packaging, and manufacturers’ stated ability to produce at least 10,000 swabs per day (at the time, roughly a week’s worth of swabs for a midsized testing center) within a week’s notice. We considered differences in supply chain to minimize the risk of future crises.

### Phase III: field testing.

**(i) Trial design and oversight.** COVIDSwab is an adaptive trial for evaluating the performance of prototypes compared to the control (see above). Participants under clinical suspicion for COVID-19 who were scheduled for standard clinical SARS-CoV-2 RT-PCR testing with a control swab were asked also to be swabbed afterward with a single prototype. Prototypes were collected in VTM in a 15-ml conical tube, transported to the clinical laboratories, stored at 4°C (same as clinical specimens), and tested until at least 10 positive and 10 negative results on control swabs were obtained (17). Sample collection was performed by trained nursing or respiratory-therapy staff (“study staff”) overseen by the respiratory therapy department at the Beth Israel Deaconess Medical Center (BIDMC). Clinical Microbiology Laboratories at BIDMC oversaw data collection. This study was reviewed and approved by the institutional review board of Beth Israel Deaconess Medical Center (protocol number 2020P000323).

**(ii) Participants.** Participants were individuals clinically suspected of COVID-19 who were brought to the drive-through/walk-up (“drive-through”) COVID-19 testing site at BIDMC. Adults over 18 years of age were given a participant information sheet by study staff and asked whether they would agree to being swabbed with a prototype swab by a trained nurse or respiratory therapist in addition to the control swab required for testing. Individuals with known thrombocytopenia of <50,000 platelets/μl were excluded from the study to avoid the risk of mild bleeding.

**(iii) Trial procedures.** Prototype swabs were individually packaged and autoclaved at BIDMC for sterilization according to manufacturer protocols. Swabbing was performed per the standard protocol. Participants were swabbed first with the control swab and then with the prototype. The choice of naris for each swab was left to study staff and the participant. Approximately half of all drive-through arrivals participated. Control and prototype swabs were placed in separate vials of VTM and transported to the BIDMC Clinical Microbiology Laboratories, where each sample was tested on the Abbott m2000 SARS-CoV-2 RT-PCR platform per the standard clinical protocol.

**(iv) Statistical analyses.** RT-PCR results are reported categorically as either positive or negative. We tested categorical concordance using Cohen’s κ value (18). For each positive test, the *C_T_* value (the RT-PCR cycle number at which the sample first turns positive) was obtained from Clinical Microbiology Laboratories. Higher values reflect lower viral load in the sample.

We tested for systematic bias in *C_T_* values by comparing values for controls versus prototypes using the Mann-Whitney U test (MWU) (19). This tested the null hypothesis that values for controls and prototypes are drawn from the same underlying distribution; a *P* value of >0.05 was interpreted as no bias. For discordant (positive control and negative prototype or vice versa) samples, the negative was assigned a *C_T_* value of 37, the total number of cycles run. As a second test for bias, we compared (again by MWU) the distribution of differences in *C_T_* values between control and prototype swabs to the distribution of differences between two control swabs taken within 24 h (quality control data independent of our study). This tested the null hypothesis that the differences between control and prototype swabs and the differences between two control swabs are drawn from the same underlying distribution; a *P* value of >0.05 was interpreted as no bias.

To quantify relative preferences among the prototypes, we performed round-robin A/B testing (20, 21). (In A/B testing, two variants of a single variable are compared and a test subject chooses his or her preference; in a round robin, a test subject has the opportunity sequentially to consider every possible pair.) Specifically, we gave each study staff member a printout of all six possible pairs of swabs, in randomized order, and for each pair asked the staff member to circle his or her preference. We collated the results and assessed preferences.

## RESULTS

### Open process.

In the first days of the development effort, a GitHub repository (10) was established to serve a public resource and knowledge base. We updated the repository continuously with design information and test results. These updates included high-resolution images of prototypes submitted to us for testing (10), a public database of results of our phase I testing, and periodic updates and guidance based on our experiences. Open communication facilitated rapid design generation by providing anyone interested with a way to quickly understand the required specifications and to learn from each other’s experiences.

### Phase I testing.

To date we have evaluated 48 materials and 160 designs submitted to us for testing by 4 individuals, 2 laboratories, and 18 companies, for a total of 24 manufacturers. Prototypes from seven manufacturers passed phase I testing. Most failures were either for inappropriate materials (too brittle, too stiff, not stiff enough, sticky, too rough), or for inappropriate designs, including those with heads that were too sharp, too flimsy, or too topologically bland (leading to relatively low surface area). Prototypes from 19 manufacturers went through at least two versions, with a maximum of 28 prototypes from one manufacturer (prototype 4; see below) (Fig. 1). The rate-limiting steps were receipt of new prototypes, with slow mail delivery during the pandemic being a major contributor, and PCR compatibility testing, as testing patient samples took priority over testing prototypes. Communication with and responsiveness of manufacturers were considered outstanding.

### Phase II and III prototypes.

Prototypes from four manufacturers have passed phase II testing (two of the seven that passed phase I are still being evaluated; one could not be manufactured at high volume), all of which have completed our phase III clinical trial: these are prototypes from the 3D-printing manufacturers Resolution Medical (with technology from Carbon3D), EnvisionTec, Origin.io, and HP, Inc. (prototypes 1 to 4, respectively) (Fig. 1a). Like control swabs, the prototypes were 15 to 16 cm in length with 1- to 3-cm-long radially symmetric heads 2 to 3 mm in diameter, a thin neck 4 to 7 cm long and 1 to 2 mm in diameter, and a thicker shaft 2 to 4 mm in diameter, with a break point most often 7 to 8 cm from the tip of the head. The materials for phase III prototypes 1 to 4 were Keysplint Soft Clear, E Guide Soft, methyl-acrylate photopolymer resin, and PA11, respectively. Head design evolved over many iterations to increase surface area. Designs generally featured either a polygonal matrix connected to a central, tapered strut with multiple branch points or else some form of spiral (Fig. 1b). Manufacturers were able to balance sample collection (Fig. 1c), stiffness, and surface texture. Variations of a longitudinal central strut allowed various degrees of stability, flexibility, and impact cushioning (Fig. 1d).

### Sample and data acquisition.

We collected and tested control and prototype swab pairs from 276 participants. VTM was used in all cases. Approximately half of the patients tested at our drive-through testing center participated. Because testing runs were batched and the COVID-19 status of participants was not known prior to testing, the number of control positives usually exceeded the minimum requirement of 10 (range, 10 to 19). Total time required for collecting all specimens for a given prototype was 2 to 3 days per prototype, and RT-PCR testing of test samples was run along with that of the clinical sample. Typically, the test sample and clinical sample were run on the same Abbott m2000 machine as part of the same batch; occasionally, a test sample was run on a different machine or in a subsequent batch, with temporary storage at 4°C. The frequency of control positive tests was 18%, generally increasing by prototype as the pandemic worsened in and around Boston.

### Comparison.

**FIG 2:**
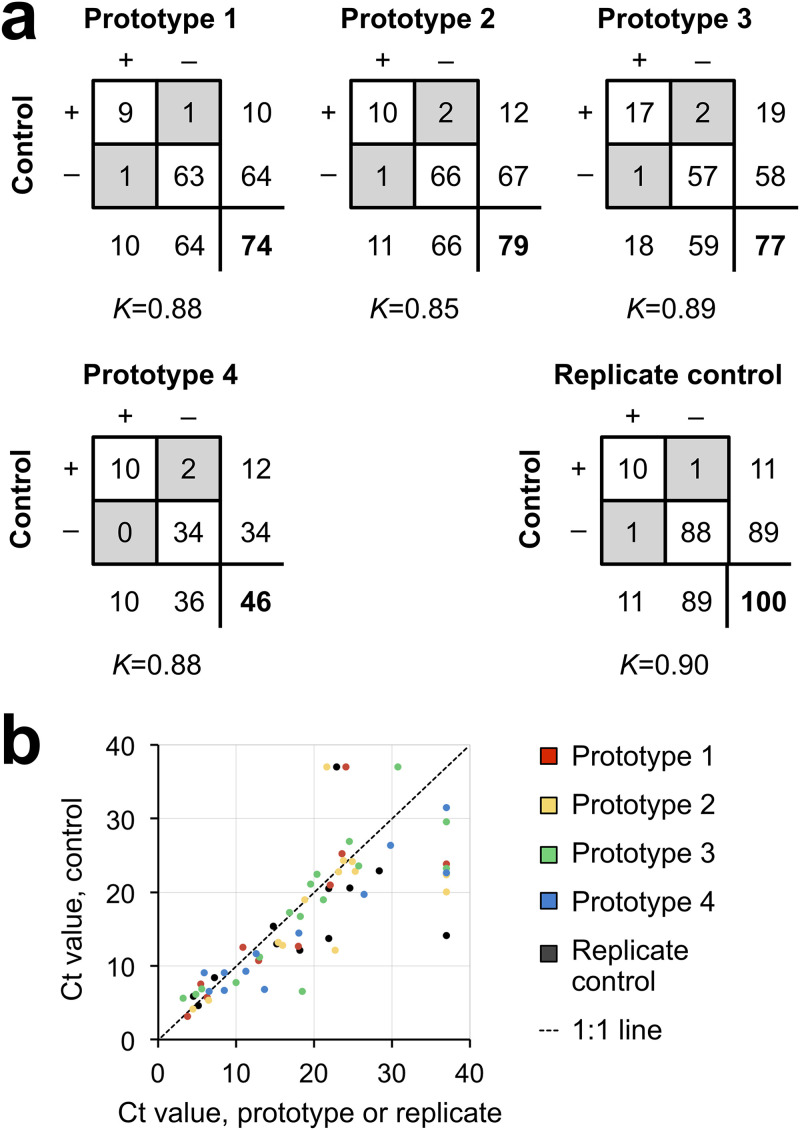
Categorical concordance versus control swab. (a) Two-by-two tables giving counts for each prototype versus the control swab and for control versus replicate control obtained within 24 h from the same individual. Discordant results are in gray, totals for each swab are below and to the right of each box, and the total number of pairs is in bold. *K*, Cohen’s kappa. (b) Scatterplot of *C_T_* values for pairs of swabs for which at least one swab was SARS-CoV-2 positive. For discordant pairs, the negative swab was assigned a *C_T_* value of 37 (the maximum number of cycles run).

All four prototypes exhibited a high degree of concordance with the control swab, with κ values of 0.88, 0.85, 0.89, and 0.88 (Fig. 2a). For convenience, we use the terminology of true positives, true negatives, false positives, and false negatives, with the control swab result considered the provisional gold standard. Prototypes exhibited 0 or 1 false positive and 1 or 2 false negatives. However, since control swabs are known to be an imperfect gold standard (<100% sensitivity) and because PCR positives are more likely to reflect true infection than error, false positives were interpreted as identifying missed infections; indeed, false positives were referred to clinical care teams as clinically actionable, as per IRB protocol. Of note, discordant cases were always associated with high *C_T_* values, reflecting low viral load (Fig. 2b). For example, for prototype 4, the control swab for one of the two false negatives had a *C_T_* of 31.47, just short of 31.50, the manufacturer’s reporting cutoff (corresponding approximately to a single virion per ml of VTM); in addition, testing of this false negative was delayed by 16 h because of prioritizing patient samples, which can result in decreased signal.

To better assess possible performance differences between control and prototype swabs, we compared *C_T_* values for control-prototype pairs in which at least one swab was positive (assigning the maximum possible *C_T_* to negatives; see Materials and Methods). Specifically, we asked whether the *C_T_* values for the prototype swabs were systematically different from those for the control swabs. Systematically higher values for prototype swabs would suggest that they may underperform control swabs, notwithstanding the high kappa values. A *P* value of >0.05 indicates no statistical difference. Although there were more data points below the 1:1 line than above it (Fig. 2b), statistical testing revealed no evidence for underperformance, with MWU *P* values of 0.36, 0.26, 0.42, and 0.31 for prototypes 1 to 4, respectively (Fig. 2b). This result supports the conclusion that the prototypes are noninferior to the control.

As an additional assessment of noninferiority, we compared the difference in *C_T_* values observed between control and prototype swabs to the differences between replicates of control swabs. Independent of our clinical trial, there were 88 cases in which a patient, in the course of clinical care, was swabbed twice within 24 h (mean ± standard deviation, 15 ± 7 h), during the time period of our study. In 11 of these cases, at least one of the two swabs was positive for SARS-CoV-2. There were two disagreements between replicate swab tests, resulting in a κ value of 0.90, similar to what was observed in our study for each prototype (κ = 0.85 to 0.89). Also as in our study, the *C_T_* values for the first swab and second swab were not significantly different (MWU *P* value of 0.18). Finally, the differences between *C_T_* values for the first and second control swabs were comparable to the differences between control and prototype swabs (MWU *P* values of 0.31, 0.26, 0.47, and 0.44 for prototypes 1 to 4) (Fig. 2b).

### Staff and participant preferences.

**FIG 3:**
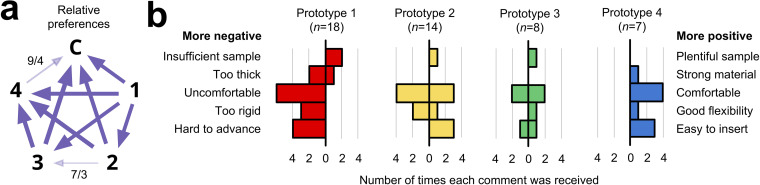
Subjective feedback. (a) Round-robin A/B testing of net preferences among prototypes 1 to 3 (large bold numbers) and the control (C). Each arrow points from the less preferred to the more preferred swab. Arrow weight indicates strength of relative preference. Preferences were unanimous except where noted with numbers separated by a slash: the first number is the number of responses for the direction indicated by the arrowhead, while the second number is the number of responses that had the opposite preference. The weight of the arrow is proportional to the difference (e.g., 7 − 3 = a net preference of 4). Unless otherwise noted, each arrow represents 12 to 15 separate responses. (b) Numbers of positive and negative comments received from study staff who administered the swabs, tabulated by category. In each plot, negative feedback is to the left of the zero, while positive feedback is to the right. The presence of bars on both the positive and negative sides of zero reflects different opinions among study staff. *n*, total number of comments received about each prototype from study staff.

A written staff survey (see Materials and Methods) showed a preference for prototype 4, then prototypes 2 and 3, and then prototype 1. There was a slight preference for the control swab over prototype 4 (Fig. 3a). In narrative feedback, prototype 4, which underwent the largest number of revisions through our process (i.e., 28), was described as comparable to the control swab (Fig. 3b).

### Availability.

Swabs are available to order. Several million have been used across the United States as of this writing. Details can be found on the GitHub repository in the updates at https://github.com/rarnaout/Covidswab/tree/master/BIDMC. Contact information for ordering can be found at http://printedswabs.org, a website set up by a consortium of academic, medical and commercial enterprises to deliver clinically tested, FDA-registered, 3D-printed COVID-19 nasopharyngeal test swabs produced at scale. Please note that this site may list manufacturers whose products were not validated in this study.

## DISCUSSION

The COVID-19 pandemic has forced health care providers to seek alternative sources of critical materials affected by supply chain disruptions and increases in demand. The situation has forced providers to innovate under extraordinary time pressure. Over the course of our study, we received numerous anecdotal reports of swab shortages at hospitals across the United States and in Europe, necessitating urgent stopgap solutions. Scientific literature on time-sensitive innovation suggests that open, collaborative, decentralized processes outperform closed or proprietary ones (11–13). Here, we report the success of such a process, going from the identification of the swab crisis to multiple clinically validated prototypes capable of high-volume manufacture beginning at 22 days. Notably, none of the prototypes tested were flocked, yet their performance was statistically indistinguishable from that of the flocked control swab.

The urgency of the situation, the configuration of the manufacturing ecosystem, and human nature contributed to several observations and shortcomings worth mentioning. First, 3D printing has important advantages in a crisis, including the ability to iterate designs and output swabs rapidly. It remains to be seen how complementary manufacturing techniques, each with advantages and disadvantages relative to 3D printing, will contribute in a more mature market and less urgent setting. Second, in any cooperative process there is a temptation to “defect,” i.e., to take without giving back. Individuals and manufacturers may well exploit open knowledge for competitive advantage (22). This is a known price of openness that can disincentivize cooperation, absent social or structural mechanisms to enforce norms; managing this temptation took considerable effort by all. Third, ideally the study would have been larger and there would have been a better null model than replicates separated by many hours to which to compare our results. Possible sources of variance in our study include differences in secretions or viral burden between nares and the possibility that the first (control) swab left less material for the second (prototype) when the same naris was used for both swabs. Despite these potential issues, our statistical tests supported analytical noninferiority for all four prototypes. Fourth, we note that our round-robin A/B testing survey was useful in summarizing the direction of preferences, although a tally of the narrative comments added useful detail regarding the strengths of the various preferences. A possible explanation is that the control swab was preferred in large part simply due to its being familiar, and it was preferred only narrowly (if often).

Like the control swab, the prototype swabs we tested can be improved upon, and manufacturers are currently doing so. The same is true for other prototypes we may test through our ongoing clinical trial. Especially in a crisis, “perfect” is the enemy of “good enough.” The pandemic continues to change quickly, and bottlenecks will likely continue to appear unpredictably. The constant requirement is the ability to respond in a timely fashion under this extraordinary pressure. We hope that our experience, based on past scientific work on cooperation and innovation, will provide a useful case study for how to design and produce a clinically validated medical device under the pressure of an ongoing pandemic, work on which others will hopefully improve as we continue to fight COVID-19 together.
